# Developing Healthcare Team Observations for Patient Safety (HTOPS): senior medical students capture everyday clinical moments

**DOI:** 10.1186/s40814-021-00891-3

**Published:** 2021-08-23

**Authors:** E. S. Anderson, T. R. L. Griffiths, T. Forey, F. Wobi, R. I. Norman, G. Martin

**Affiliations:** 1 College of Life Sciences, Leicester Medical School, Leicester, UK; 2 Leicester Medical School and Consultant Urological Surgeon at University Hospitals of Leicester NHS Trust, Leicester, UK; 3 ReSET, IT Services, University of Leicester, Leicester, UK; 4 Health Sciences Department, College of Life Sciences, Leicester University, Leicester, UK; 5 The Healthcare Improvement Studies Institute, Clifford Allbutt Building, Cambridge Biomedical Campus, Cambridge, CB2 0AH UK

**Keywords:** Observation of practice, Light noise, Good practice, Poor practice

## Abstract

**Background:**

Aviation has used a real-time observation method to advance anonymised feedback to the front-line and improve safe practice. Using an experiential learning method, this pilot study aimed to develop an observation-based real-time learning tool for final-year medical students with potential wider use in clinical practice.

**Methods:**

Using participatory action research, we collected data on medical students’ observations of real-time clinical practice. The observation data was analysed thematically and shared with a steering group of experts to agree a framework for recording observations. A sample of students (observers) and front-line clinical staff (observed) completed one-to-one interviews on their experiences. The interviews were analysed using thematic analysis.

**Results:**

Thirty-seven medical students identified 917 issues in wards, theatres and clinics in an acute hospital trust. These issues were grouped into the themes of human influences, work environment and systems. Aviation approaches were adapted to develop an app capable of recording real-time positive and negative clinical incidents. Five students and eleven clinical staff were interviewed and shared their views on the value of a process that helped them learn and has the potential to advance the quality of practice. Concerns were shared about how the observational process is managed.

**Conclusion:**

The study developed an app (Healthcare Team Observations for Patient Safety—HTOPS), for recording good and poor clinical individual and team behaviour in acute-care practice. The process advanced medical student learning about patient safety. The tool can identify the totality of patient safety practice and illuminate strength and weakness. HTOPS offers the opportunity for collective ownership of safety concerns without blame and has been positively received by all stakeholders. The next steps will further refine the app for use in all clinical areas for capturing light noise.

## Key messages regarding feasibility

- What uncertainties regarding feasibility existed prior to this study?

It remains difficult to teach undergraduate healthcare students about the realities of patient safety in complex every day clinical situations. We set out to find a way to address this problem and turned to aviation, where anonymous observation of the crew has advanced safe practice. In this study, we present work to make this a reality for final-year medical students, mindful of acceptability of being an observer and being observed.

- What are the key findings on feasibility from this study?

Using cyclical action research over several years, we designed an intuitive app for medical students in their final year. Drawing on expertise from aviation and having a wide stakeholder steering group was important as the group received staged outcomes and guided the study. Medical students in this study helped to translate the aviation approach into a method for student learning with opportunities for front-line staff to reflect on their practice. We adapted the aviation methodology for healthcare and moved from a paper data collection tool to an easy-to-use app. We now have a usable observation tool to appreciate the complexity of patient safety. We learnt that it was acceptable to observe good and suboptimal practice.

- What are the implications of the findings on the design of the main study?

The methodology helped to adapt thinking from aviation to healthcare, but we have work to do on culture change for the acceptability of anonymous observation in all healthcare arenas. Building on this study, we aim to further pilot and test the use of the app in more clinical areas using training and preparation sessions for acceptability by all clinical staff.

## Background

In the last decade, the analysis of patient safety events has led to the identification of circumstances which contribute to safe and unsafe patient care [1]. These include not just active failings, but a wider range of more latent influences such as human factors, systems, aspects of the environment and poor professional practice. Interventions to address these contributory factors have led to some reduction in errors but high levels of concern remain [2–6]. The analysis of data after safety events has been helpful, although accurate recording and understanding of the totality of patient safety data remains a challenge, as busy practitioners fail to report events, often because they are not shown the benefits [7–9]. There are still big questions to be addressed about how to make changes that will advance standards for safe effective care [10].

Mastering the complexity of healthcare delivery remains tortuous. Hard data for accountability purposes highlights variability in one area of practice at one moment in time but fails to assimilate and understand the integration of care delivery across all levels of an organisation [11]. It often fails to pick up the nuances involved including the patient and practitioner perspectives. Triangulation of data to include patient and staff perceptions may offer a deeper understanding because it values users or front-line stakeholders as owners of the standards of their work [12]. Findings from large-scale studies reveal that front-line practitioners need the right resources, including staffing levels, support and encouragement from leaders who are collaborative, values-based and uphold person-centred care [13]. Practitioners also need simple holistic feedback about ‘uncomfortable information’ and the ‘blind spots’ that need to change [13, 14]. Data collection that is not meaningful to healthcare staff at all levels of an organisation can generate suspicion and resistance and militate against the aspirations for building a safe culture for healthcare delivery [15]. Calls for the next steps for patient safety seek greater clarity about how to identify and measure hazards in real time to intervene before incidents occur [10] and to focus more attention on what works well learning from good practice [16, 17]. Moving from the reactive focus on the negatives, what goes wrong, towards a proactive focus on good practice requires a different approach to seeking and learning from clinical successes. Positive deviance highlights the success of individuals, teams and organisations and seeks to aspire others to adopt positive solutions to achieve effective safe practice [4]. This requires transparency and willingness to share and for others to adapt and adopt tested work patterns within their locality [11].

Potentially valuable sources of knowledge, about the good and the potentially problematic features of care, are groups such as junior clinicians and medical students, who experience multiple settings and so have potentially unique comparative insight. The Francis and Keogh reports both suggested a special place for junior doctors and student nurses in identifying differences in standards of care between the organisations employing them, based on their simultaneous position as insiders and outsiders within the system [18–20]. Ladden and colleagues considered the place of students who stand outside and yet are within everyday clinical practice and from this unique vantage point perceive what is going on: ‘Ask any medical resident or graduate nursing student working on the front lines of care about quality and safety problems and you had better be prepared to listen for a while. What they see is what we all read about: serious shortcomings in our systems of care — delays, errors, confused families, and daily workarounds to get patients what they need. Despite their front-line view of the problems, learners are almost never involved in workplace-based experiences to learn about systems improvement’ [21]. Only recently have healthcare curricula been expected to deepen student understandings of patient safety at pre-registration/undergraduate level [22–24], despite calls from the World Health Organization who have designed a patient safety curriculum guide [25, 26]. The General Medical Council (GMC), in asking for more teaching on patient safety, has highlighted some medical school programmes where students have been asked to observe what is happening in everyday practice [27]. Despite this, many medical students remain unfamiliar with the scope of learning for safe practice [28]. Transforming healthcare education and equipping students with the right approach is seen as one of the essential changes required, if we are to change culture and advance safe practice [10]. An untested route to identify both good and poor quality care could emerge from proactive students [29]. Students observe poor practice [30] and could also anonymously highlight hazards in everyday practice and simultaneously advance their learning concerning patient safety.

### Background to the study

Looking to advance medical student understandings of patient safety and aware that they are observers of the system, we were drawn to observation approaches used in aviation to inform best practice. Adopting learning from high-risk industries for patient safety has already taken place within healthcare delivery [31, 32]. Our journey of discovery started with an aviation strategy to identify the contributory factors which underpin human error. This strategy known as the line operations safety audit (LOSA) is used to collect data about flight crew behaviour and situational factors on flights. The first audit was developed in the USA and has been internationally adopted [33, 34]. The findings collected over time have illuminated how people behave in real-time, offering evidence to improve safety [35, 36]. The attractions of this real-time observation reporting are that the data are collected prospectively and anonymised, with the results fed back to help change practice. This work has contributed to the flattened non-hierarchy team-based culture that characterises modern aviation [37]. While acknowledging important differences between civil aviation and healthcare that are sometimes overlooked in efforts to import safety interventions from one context to the other [38], we nevertheless saw potential in adapting this approach with a view to both identifying influences on healthcare safety and enriching undergraduate education. We outline the process of adaptation for medical student learning.

## Methods

### Aims and objectives of the study

This exploratory research aimed to develop an observational recording process for patient safety learning by final-year medical students. The study research protocol was funded, in September 2018, by the University of Leicester Wellcome Trust, Institutional Strategic Support Fund (grant RM32J0012M3). The bid aimed to build on our understandings of LOSA as a self-administered (organic), proactive risk-management tool that records ‘Threats’ and ‘Errors’ (International Civil Aviation Organisation, 2002) to develop a usable healthcare normal operations safety audit intervention. There were two intended outcomes from the study: (1) to produce a learning tool for educating medical students in patient safety and (2) to design a patient safety risk-management tool. The development was conceptualised as a staged process. We report on the first stage only.

### Study design

The design involved the cyclical collection of student observational data over three iterations, following the principles of participatory action research (PAR) [39], to reach a pragmatic approach for healthcare similar to that used in aviation [40]. In this mixed methods design, the researchers’ understanding of the social setting was developed through the data generated by participants (including participating students’ observations of clinical practice in real-time, and the reflections of those involved in the process); this improved understanding then contributed to further improvement of the intervention over the course of the study [41].

### Participants

This iterative process involved a research partnership between the stakeholders and researchers, overseen by a steering group (patients, students, an airline pilot, academics, clinicians, local patient safety leads). The steering group met twice annually, before and after student observations.

The data collectors were final-year medical students. We saw this group as particularly appropriate for the project, since they have undertaken substantial clinical training and so have the ability to understand the constituent parts of good practice, but are not yet embedded in any healthcare organisation, and so retain the perspective of an outsider. There was only one access point to final-year medical students in the annual spring special study module (SSM). Here, students chose from a range of learning possibilities to enhance their abilities to work as junior doctors. This project was submitted as a SSM and attracted students who selected the project annually over the 3 years.

### Data collection

Data were collected iteratively over 3 years, following the schedule of final-year medical students completing the SSM. Prior to data collection, the student participants were prepared for their role as researcher data collectors. They spent 1 day reflecting on their learning on patient safety including human factors facilitated by experts. The second day was spent learning about being an observer researcher and becoming familiar with the data collection tools. In the first two cycles of data collection, recordings were made using paper grids which evolved and changed over time. In the third and final cycle, data were collected using the first version of an app developed based on experiences in the first 2 years. Students were asked to record what they saw using scales and with written detailed comments. This year-on-year learning across successive student cohorts enabled changes to be made and in this way the student observations and interactions with real-time clinical events shaped the design of the final app-based data collection mechanism. As the SSM covered 3 weeks, students were able to spend up to 9 days observing, the remainder of their time was spent in feedback and training.

### Data analysis

The student scored and written observational data was analysed by the academic researchers (ES and LG) for clarification and agreement with individual students halfway through the learning placement, to identify problems and issues (e.g. inconsistencies, lack of clarity) in recording their observations. At the end of their observation period, students presented their findings for the first time to the clinical staff and senior patient safety leads in the organisation where they had undertaken their observations. The recorded observations were collected and analysed for common themes using the principles of thematic analysis (ES and LG) [42]. The scoring scheme evolved over time from scales of 1–5 indicating the severity of poor practice to a scale which graded both good and poor practice using two levels each, supported by event descriptions.

Ward and other clinical areas covered by the study were selected by the steering group partnership, which involved local hospital patient safety leads and clinicians who were medical educators and familiar with patient safety. The academic clinical leads briefed and prepared the clinical areas to receive the students. Wards, clinics and operating theatres were involved.

In the last cycle, all stakeholder perceptions and experiences of the process were collected using one-to-one semi-structured individual interviews with medical students (observers) and clinical teams (observed practitioners), using an independent researcher (FW) who was not known to the medical students. The interviews continued until theoretical saturation was reached. Interviews were guided by a topic guide focusing on the experiences of both the observers and the observed. With the consent of the participants all interviews were audio recorded and later transcribed and analysed using thematic analysis. In this way, the experiences of the key stakeholders were clarified.

This study received ethical permission from University of Leicester (7741-esa1-medicaleducation).

## Results

**Fig. 1:**
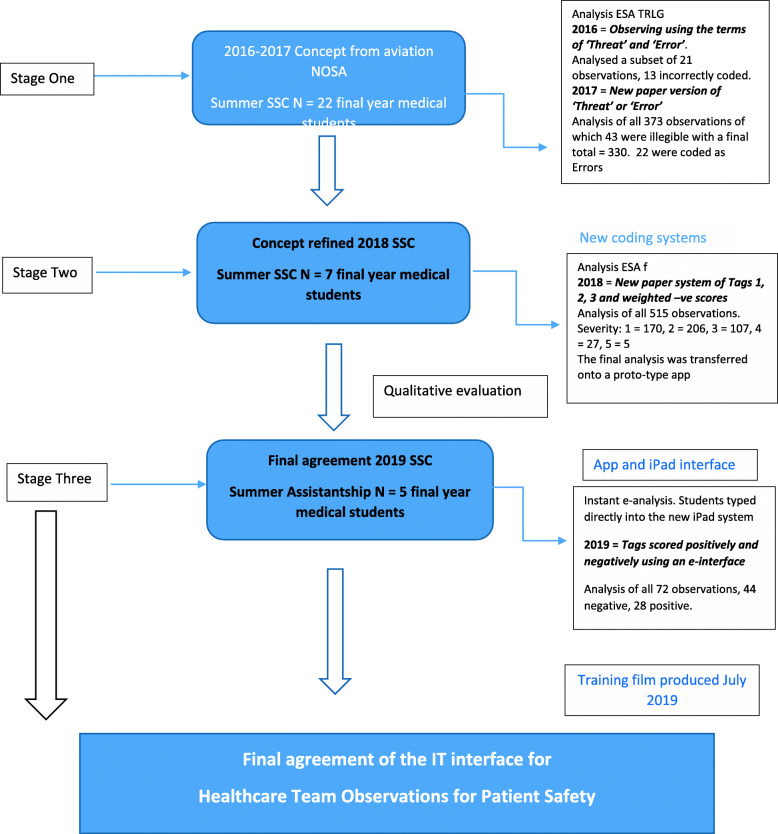
Time line of the development of Healthcare Team Observations for Patient Safety (HTOPS)

We present the development of the Healthcare Team Observation for Patient Safety (HTOPS) platform and process chronologically over three stages; the timeline can be seen in Fig. 1. The first stage combines the first two cycles of learning as this was an exploratory phase. The work was refined over 3 years, and we reflect on key learning points that fed into the development and refinement of the system.

### Stages of the observation tool development

#### Stage one (2016–2017)

The adaptation of the aviation observation process to identify patient safety concerns started with the final-year special study module (SSM) in 2016 and 2017 (*n* = 11 in each cohort; total *n* = 22). We started with aviation terminology, namely ‘Threats’ observed in the working environment and ‘Errors’, i.e. perceived noncompliance with rules/policy/guidelines. In discussion with a clinical team (senior nurse and consultants from a local hospital), a set of possible healthcare threats and errors was agreed through a brainstorming exercise. They included a list of possible ‘Threats’ relating to human factors, technology and building/environment. The ‘Error’ list included noncompliance with rules relating to prescribing, ordering investigations and their interpretation, and patient and practitioner communication. We gave a code number to each possible threat and error. The students were asked to complete observations in a range of clinical areas: two operating theatres receiving for orthopaedic and urology, outpatient fracture clinics, ante-natal wards and clinics, and medical wards. Students were given training on patient safety, observation techniques and the coding system template with the threat and error code list to record what they observed during a session (morning or afternoon) (Table 1). The students spent 6 days observing clinical practice moving between their allocated wards, theatres or clinics.

**Table 1:**
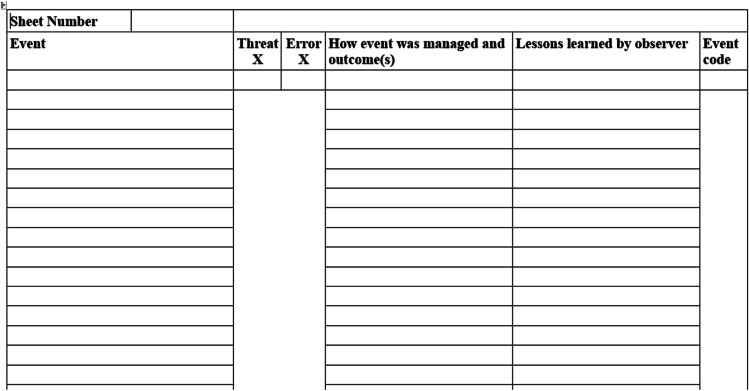
Stage one: design for recording observations (pilot 2016)

#### Evaluation outcomes

In 2016, students recorded a large number of observations and we analysed a subset of the data (21 scripts) for checking the process (Table 2). We found that students confused threat and error in 13 instances. We refined the paper recording sheet to enable students to write more narrative to justify their findings. In 2017, we analysed all the outcomes from the student observations (*n* = 373 observations); 43 were illegible and were withdrawn, leaving 330 for full analysis of observed care practice on wards, clinics and theatres, of which there were 22 errors (Table 3). Students continued to have difficulty in differentiating between threats and errors in the midst of the complexity of everyday clinical practice and found the codes cumbersome. However, they were able to step back and observe care delivery in real time, noticing a plethora of concerns relating to both sloppy practice (e.g. hand hygiene) and systems issues (e.g. caused by poor geographical layout). All students reported they had advanced their understandings of patient safety. The observations revealed the students’ lack of familiarity with the setting helped in identifying features that seemed inappropriate, whereas practitioners around them had normalised these practices. Some errors reported were incorrect and misleading, reflecting students’ unfamiliarity with speciality-specific safe practice.

**Table 2:** Stage one 2016 examples (subset of 21 observations analysed 13 incorrect codes)

Code theme	Description	***E***rror or ***T***hreat	Student correct in E or T	Number
**Infection**	• Consultant not washing hands between patients	T	Incorrect	1
	• Failure of medical staff to change uniform/dress is a threat to other patients	T	Correct	1
**Insufficient skills**	• Operation where staff had not recognised the drill setting before starting	T	Incorrect	1
**Wrong patient**	• Similar patient’s consultant confuses the patients corrected by Registrar	T	Incorrect	1
**Privacy and dignity**	• Curtain not fully pulled around patient during a ward round	T	Incorrect	1
	• Sensitive information spoken loudly at the ward desk	T	Incorrect	1
**Slow computers**	• Issues with slow computers	T	Correct	1
**Technology**	• No dicta-phone available	T	Correct	1
**Layout design**	• Layout design of clinic	T	Correct	1
**Equipment**	• Needed help to identify the right equipment before surgery—pieces missing	T	Incorrect	1
**Guidelines not followed**	• Changing uniform/clinical dress in isolation wards between seeing different patients	E	Undefined	1
**Poor professionalism**	• Management of discharge—varicose veins	T	Incorrect	1
**Confidentiality and Patient-centred care**	• Junior doctor dictating notes with door open	T	Incorrect	1
**Checking**	• Patient who was not sent his operation date—took a year—administrative error	T	Incorrect	1
**Systems issues**	• Transferring data from paper to IT prescribing	T	Correct	1
	• The white board was not up dated at handover	T	Incorrect	1
	• Bed shortages and problems with transfer of patient back to ward from ITU	T	Correct	1
**Team communication**	• Junior doctor not prepared for the ward round and had to go back and gather more data	T	Incorrect	1
	• Nurse joining a ward/team meetings out of sequence with which patient is being discussed and wrong information given corrected by consultant	T	Correct	1
**Excluded data wrongly included neither error or threat**				
**Error or Threat**	**Issues raised**			
**T**	Recorded as slow computers, whereas the installation of dictate software by a new clinician is a normal process. If we had had a chat as a team, this understanding about a normal processes could have been relayed.			
**E**	Transfer of patient to the ward during the night from ITU is normal practice and within hospital protocol

**Table 3:** Cohort 2017: summary outcomes (total 330 of which 22 errors)

Code theme ***E = error****(numbers in italics)* T = threats or concerns	Description
**Error for infection-relating to staff** *E = 8* T = 44	**Errors** • Nurse removes clips wearing gloves but bin not working so touches with her gloved hand and then checked patient wound (× 2). • Consultant on the phone in personal protective equipment (PPE) leaves isolation room still wearing PPE (× 2). • Operating department practitioner started an incident investigation for non-sterile equipment in theatre (× 2). • Patient had diarrhoea for 3 days and no stool sample taken. • Failure to gown up properly in an MRSA area—infection control told. **Other examples of threat concerns** *Not hand washing* • Doctor did not wash hands before entering the bed space and examining the patient. • Ward round no one washed hands between patients. Practitioners coughing, sneezing • Consultant sneezes into hands and proceeds to touch iphone, obs chart and patient’s bedside. No equipment to support handwashing • No alcohol hand gel in bay areas to wash hands. • Physio equipment blocking access to hand gel—no hand washing. *Poor infection control awareness* • Theatre staff repeatedly brushing against non-sterile parts of the theatre.
**Staff related** T = 16	Stretched Staff • Junior doctor in a hurry left with two pagers when on call. • Registrar taken from ward round but knowledge was vital. • Too many patients on a theatre list.
**Health and safety** T = 4	• Sharps bin not secure. • Wet floor in theatre not wiped.
**Patient notes** T = 10	• Midwife unable to read doctors writing. • Patient could not be discharged as notes missing.
**Similar patient names** *E = 1* T = 4	**Error** • Wrong patient—brought one with the same name.
**Laterality** T = 1	• Incorrect limb labelling.
**Privacy, dignity and confidentiality** *E = 1* T = 45	**Error** • Patient not involved in consultation. All advice and explanations were given to the relative; there were no mental capacity issues. Lack of patient involvement meant no ability to raise concerns. **Other examples of threat concerns** • Discussing patient in a corridor patient overhears and states ‘that’s me’. • Finally a scrub nurse covers patient but left exposed unnecessarily. • Imaging software prompts for password after user name entered.
**Computer related** T = 24	• Had to get x 3 computers to try and access a radiology image. • Ward round delayed as no portable computer.
**Escalation and patients waiting** T = 2	• Patient no referred quickly from A/E and could not receive care available—cause of delay unknown.
**Risks from poor practice** *E = 1* T = 2	**Error** • X-ray showed patient had a bracelet on under her plaster. **Other examples of threat concerns** • Cast removed and pressure sore apparent.
**Language** T = 10	• Mother with no English did not know to prepare child for pain on removal of K-wires. • Deaf patient had to rely on lip reading.
**Not thinking** *E = 2* T = 9	**Error** • WHO check list all done from memory (× 2). **Other examples of threat concerns** • Removal of K-wires form child without checking how many were there. • Junior doctor reads patients S number from memory. • Imaging not consulted before K-wires removed. • Not checking who was in the clinic assumed it was the husband. • Patient in fracture clinic can be with nurses, X-ray etc. and there is no record of where they are.
**Team issues** *E = 5* T = 24	**Errors** • White board recording swabs and operation equipment was wiped clear before final count and end of operation. • Trainee junior doctor missed the introductory huddle and WHO theatre check and goes onto conduct procedures despite not knowing the team and what was going on. • Breakdown in communication advanced nurse practitioner in the community had left bandages on too long—poor dialogue between the teams. • WHO checklist not read out and considered in theatre (× 2). **Other examples of threat concerns** • Team briefing using a structured check list was interrupted by midwives swapping places so neither heard the entire brief. • Poor communication between doctor and nurse, ‘Nurse you sort this out’ what? • Use of jargon in a team juniors did not understand.
**Environment/design** T = 40	• Clock incorrect in theatre. • Poor place for discussion and group huddle were interrupted with people walking through. • Fracture clinical no space for people in wheelchairs. • Clinic so hot pregnancy mothers have fainted. • Physiotherapists and plaster technicians share a room—no privacy and plaster equipment a hazard for the patients and physios.
**Drug related** *E = 3* T = 4	**Errors** • Patient left on a medicine (Tamsulosin) after a Transurethral Resection of Prostrate when no longer needed. Patient now come in for a cataract operation—error noticed. • Junior Doctor prescribed the wrong dose the pharmacist corrected before administration. • Drugs drawn up and forgotten about the consultant anaesthetist notices and asks the core trainee what it is for and gives the drug—poor communication chatting. **Other examples of threat concerns** • Cannot read prescription.
**Recording clinical information and consent** T = 5	• Foetus scan incorrect. • Consent where anaesthetic risk not mentioned and patient given unclear information about operation risks.
**Investigation related** *E = 1* T = 4	**Error** • Radiology error: In this case patient had a ring block for manipulation of a fracture but it was not a fracture. **Other examples of threat concerns** • Insufficient X-ray view obtained.
**Equipment related** T = 10	• Wrong bed type for operation—had to be changed. • Use of a radiator for placing equipment as not enough space or trolleys in the room.
**Poor professionalism** T = 21	• Registrar answers a phone inform of patient and walks out no explanation. • Patients notes not in trolley—they were in the wrong slot—took 4/5 staff several minutes to find them—but easy to put back in the right place. • Radiographer and ODP talking and joking over a patient under local anaesthetic.
**Checking** T = 9	• Points for patients ID × 3 point check. • Wrong patients imaging results checked but was spotted long chain of repeating numbers… too many digits. • Missing test results. • Difficult to read handwriting.
**Organisational issues** T = 4	• Clinic too many doctors today but the other day too few—disorganisation of the clinics. • Multiple copies of a patients notes—they had been duplicated. • Organisation of notes from trolley to desk in outpatient clinic chaotic. • Only one key to medicines cabinet—could not find nurse with key.
**Distraction** T = 18	• Lots of background noise while WHO check list being done—not everyone could hear. • Registrar having to leave patient on several occasions to take phone calls. • Consultant writing notes frequently interrupted with questions. • Wrong notes brought to patient room. • Lack of read back different members of the team meet and are not discussing the same patient—different S numbers. • Towards end of surgery lots of different conversations going on at once—staff just focussing on individual tasks this is probably because it has been a long procedure.

The evaluation in 2016 and 2017 led us to the conclusion that the categories of threat and error were too simplistic to capture the complexity of healthcare environment and care delivery (Table 4).

**Table 4:** Complexity of healthcare. The complexity issues when comparing safety between healthcare and aviation

Comparators	Aviation issues	Healthcare issues
The environment	The airplane structure varies only slightly between models—mostly this is about size. The cockpit is separate and distinct from the main part of the plane accessed by the public. In large airplanes, there is a separate staff area.	Healthcare delivery takes place in the community and in hospitals. In the community primary/family medicine is available in health centres. Hospitals consist of outpatient clinics, wards, high dependency/intensive care units, emergency care and specific care sites such as maternity. Clinical units for procedures and theatres for operations; these areas have pre- and post-assessment zones. There are specific staff areas/rooms.
The workforce	The main workforce on the airplane includes the captain and his deputies from one to a possible 4 others and cabin crew from one to a maximum of 27. There is also the ground staff at airports who welcome passengers and those who supply and support the mechanics of the airplane and equipment and resources for passengers.	The healthcare systems globally employ a vast range of staff. Trained healthcare professional includes doctors, nurses, pharmacists and allied health professions in a range of different positions of responsibility. These professional practitioners work with a plethora of support workers from receptionist to administrators, scientists and many others. Health care involves social care, policing, housing and other external support public organisations including the voluntary sector, families and/or carers.
Working timeframes	Air transport offers travel from place A to place B. This line of flight never varies, unless in an emergency, although each route is different and each airport. The line is always the direction of travel. The main risks are take-off and landing but can include incidents during or before and immediately after flight.	In healthcare, patients may have similar conditions but there is never a clear pathway or journey of care for any one individual as everyone is different. No two people are the same and no two families/carers. The possibilities are endless for involvement of different practitioners and support staff.
Policy	Set and adhered to and overseen by a few international or national governing bodies including International Civil Aviation Organisation (ICAO), European Authority in Safety Aviation (EASA) and the UK Civil Aviation Authority (UKCAA).	Each country operates different healthcare systems some in the public domain (the NHS in the UK—small private sector) some mainly private (USA) and others are a mix of public and private. Policies vary between systems and within systems. In some systems, each hospital or unit runs its own policies, which remain local to them.

We revised our categorisation framework to reflect clusters of themes identified in our analysis of the real-time student observations. There were termed tags, as follows (Table 5):

**Table 5:** Tagging framework HTOPS 2018

Tag 1: Human influences (including physical and emotional behaviour)	
**Action**	Something you do not do or should not be doing and as such is an inappropriate action e.g. a procedure like handwashing. Propping a door open when it should be shut, needle stick injury, not putting sharps in the bin properly
**Complacency**	FYI read patient S number from memory, taking short cuts, e.g. being in a rush to go to the next patient in a ward round
**Communication**	**Practitioners:** Cannot read handwriting, wiping the theatre board of patient data before theatre finished, poor communication between doctor and nurse, poor introduction(s), poor incomplete handover and manipulative communication **Patients:** Checking for ID/confirm who you are, incorrect name above the bed
**Confidentiality**	Leaving computers open and unattended, leaving notes unattended. Talking about patients in public spaces with colleagues, or in-front of patients
**Dignity**	Leaving curtains open, patient exposed, patient hurt or caused some type of distress, patient pulled backwards in a wheel chair, patient referred to by room number condition, as ‘him’ or ‘her’
**Distraction**	Something or someone who is distracting another, interruptions
**Doing the Job**	Prescribing, consenting, ordering, swab count, unlabelled specimen container, not writing clearly in the notes, using not up to date results, failure to ask about pain
**Hygiene**	Infection control and risk—e.g. hand washing
**Language**	**Practitioner** e.g. foreign, using jargon, abbreviations **Patient** e.g. foreign, deafness, disability dementia, disregard to cultural needs
**Leadership**	Self-awareness, awareness of others, ability to praise or admonish another. Leadership in collaborative patient-centred teams. Poor involvement of the patient in the team
**Privacy**	Overhearing private conversations, patient in ward hears what is being discussed at the ward desk
**Professional**	Decision making e.g. failure to use correct medication (lack of knowledge, missed allergy, wrong dose), medical interference e.g. unnecessary procedure test completed, not stopping a medication during surgical pathway; being unkind to a colleagues with loss of self-control; not acknowledging and listening to patient worries; and lack of patient centredness. Civility, respect and responsibility for personal health.
**Situation awareness**	Definition = ‘Our mental picture of what is happening around us and what is about to happen’ (Mitchell, pg. 27). See issues about an individual or about the environment around you or the systems you are using. Wrong site for surgery. Staff complaining about their work life to patients. Counting swabs in memory not aloud
**Skills**	Insufficient experience
**Stress and fatigue**	Exhausted staff who are complaining they are not having breaks and short cuts taken. Staff, volatile emotions and poor moral
**Team functioning**	Clearly not relating to everyone in a team. Team fails to connect with one another; missing member of the team.
**Tag 2: Work environment**	
**Equipment**	No BP machine. Appropriate specialist equipment not available or working. Wet plaster left on floor, phone issues, missing items in a theatre-pack
**Hardware**	Furniture, physical component of a building and how it is cared for air conditioning, cleanliness, door propped open, wet floor, endoscope issues
**Layout/design**	Seating, inadequate meeting space, wrong bed type, no wheel chair space
**Software**	IT—computers and software used to support clinical activities, slow computer responses, not enough, no Wi-Fi on the ward, mouse not working, electronic prescribing issues, not logging off computer and ICE not working
**Tag 3: Systems**	
**Checking lists**	Patient ID, WHO check list and check lists
**Equipment**	Repairs and maintenance. How failures or broken things are fixed, charging of electronic equipment
**Hand overs**	Lost drug keys
**Insufficient staff skill mix**	Too few staff or too many and inappropriate level of staff. Insufficient staff, no breaks as not enough staff, and insufficient skill mix. Change over doctor, induction and support for any staff.
**Management/leadership**	Ordering materials for a ward/unit. Staff rota, clinic set up, are asset up. Access for personal log in. Ordering materials and equipment. Who types us notes and who knows. Case selection for the right clinician.
**Records**	Documentation/forms. How notes are accessed or made available; Appointments and how they are made; multiple copies of patient notes
**Transport**	Ambulance and taxi issues

*Tag 1, human influences*. The interactions amongst humans, ‘what I do when I am with others’ and other aspects of healthcare delivery. This includes the way in which one acts or conducts oneself professionally with patients and staff and individuals’ physical actions performed incorrectly or not completed.

*Tag 2, work environment*. Relating to the physical layout/style and content within the building

*Tag 3, systems*. Things or parts that function together. The way humans interact with the environment including the level of staff required to function adequately to manage the clinical area

To help identify the level of concerns, each tag was awarded a score on a scale from 1 (a little concern) to 5 (a great deal). In addition, the tag could relate to an individual scored as one person (A—alone) or for practitioners working together in a team (T = team) of practitioners. At this stage we left these senior students to allocate the weight of concern following their patent safety training which explored never events and serious incidents.

#### Stage two (2018–2019)

In 2018, seven students used the revised paper recording system and worked in pairs in clinical areas. Of the 638 recorded tags, 123 duplicates (students recording the same observation) were removed. At this time, a new electronic database for recording the data was completed and these 515 safety concerns were transferred to the electronic system. These recordings contained 170 scores rated as ‘1’ (low concern), 206 as ‘2’, 107 as ‘3’, 27 as ‘4’, and five scored as serious, ‘5’ (a great deal). The five serious tags were all human influences (tag 1):

i. *Complacency — action*. Anaesthetic drug not labelled during a spinal epidural
ii. *Confidentiality*. Computer system open with patient results for everyone to see
iii. *Action*. Sharps not disposed of correctly during a procedure
iv. *Team functioning*. Surgical whiteboard incorrect documentation of use of needles during surgery
v. *Team functioning*. Change in surgical list led to preparation in theatre for the wrong patient

**Fig. 2:**
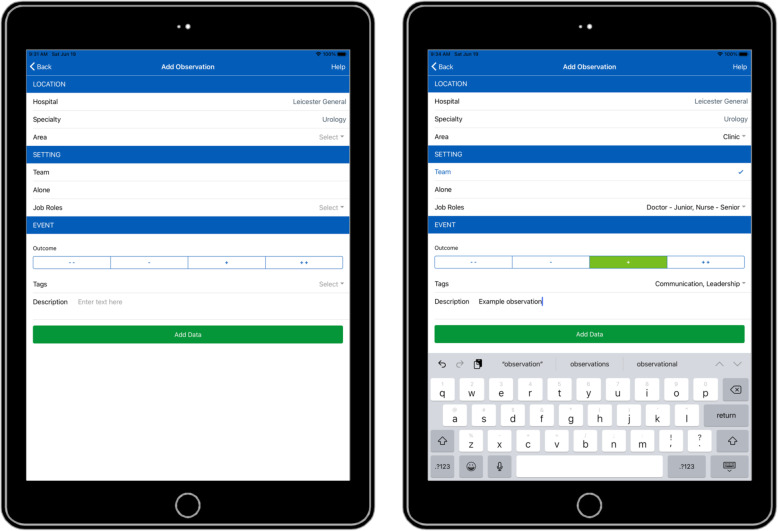
Screenshots of the input form within the app. This shows the input form within the app, both before and after completion. **A** This insert shows how the data can be presented graphically to total the number of observations by type from positive to negative. **B** This insert shows how the data can be presented graphically listing the person observed by type. The colour code show the number of this time a particular practitioners were observed and the type of observation from positive to negative. **C** This insert shows how the data can be presented graphically by colour code showing the descriptor — these can be opened on the app to show the detailed description

The concerns from this analysis revealed that it was hard for students to rate the severity of patient safety concerns on a five point scale. For this reason it was decided to reduce the weight of the scale to *two points*. The steering group reflected on the student feedback and realised that students were also verbally reporting seeing positive behaviour, which the recording system did not allow them to record. It was therefore agreed to capture all that students were seeing and record observations of good practice, resulting in a scale that incorporated two negative (− 1 and − 2) and two positive (+ 1 and + 2) scores for the new app (Table 6 — app design) (Fig. 2).

**Table 6:** App design

Field	Input	Design decision
Hospital	Select a single hospital name	As this is unlikely to change between observations, this only needed to be set once per session.
Speciality	Select a single speciality/department	As above, only set this once per session.
Area	Select between clinic, ward, theatre, pre/post-theatre and other	This selection needed to be made on each observation as it is likely the observer would move around between areas.
Setting	Select either team or alone	Quickly (single click) select whether it was observed in a team or alone setting.
Job Role	Select one or more job roles	We had a broad list of possible job roles that observers could select all the roles involved.
Outcome	Select either strong negative (−−), negative (−), positive (+) or strong positive (++)	By including positive observations, we provided a smaller and easier to use scale of outcome.
Tags	Select one or more descriptive tags	Similar to the job roles, this was a broad list of tags that one or more could be selected to describe the event.
Description	Free text input	The free text input is the slowest input, but is required to clarify the observation.
**Additional information**		
● The input form was designed to be quick and easy to use, while also gathering as much information as possible. ● The app was created as a hybrid application using the ionic framework and can therefore run on both Android and iOS devices. ● The options for the hospital, speciality, job role and tag fields are stored in an SQL database on the server, which the mobile app accesses via an API on first logging in. This means that the options can be edited at any point without having to update the app. ● To further speed up the data input, an observation can be incremented if it is observed more than once. Also, each entry can be edited on the device if the observer ran out of time to enter all fields in one go. ● Once the user is ready, all results on the app can be uploaded to the server, this requires confirming the username and password are correct. Once uploaded, the observations are deleted from the device. ● As the mobile app only connects to the server API on initial login and when uploading observations, the majority of the time it will function offline—storing the data in an SQLite database on the device. ● As well as needing a valid username and password to connect to the server, the app also required the device to have a secure pin set.

#### Sub-group 2018/2019 — new app

**Fig. 3:**
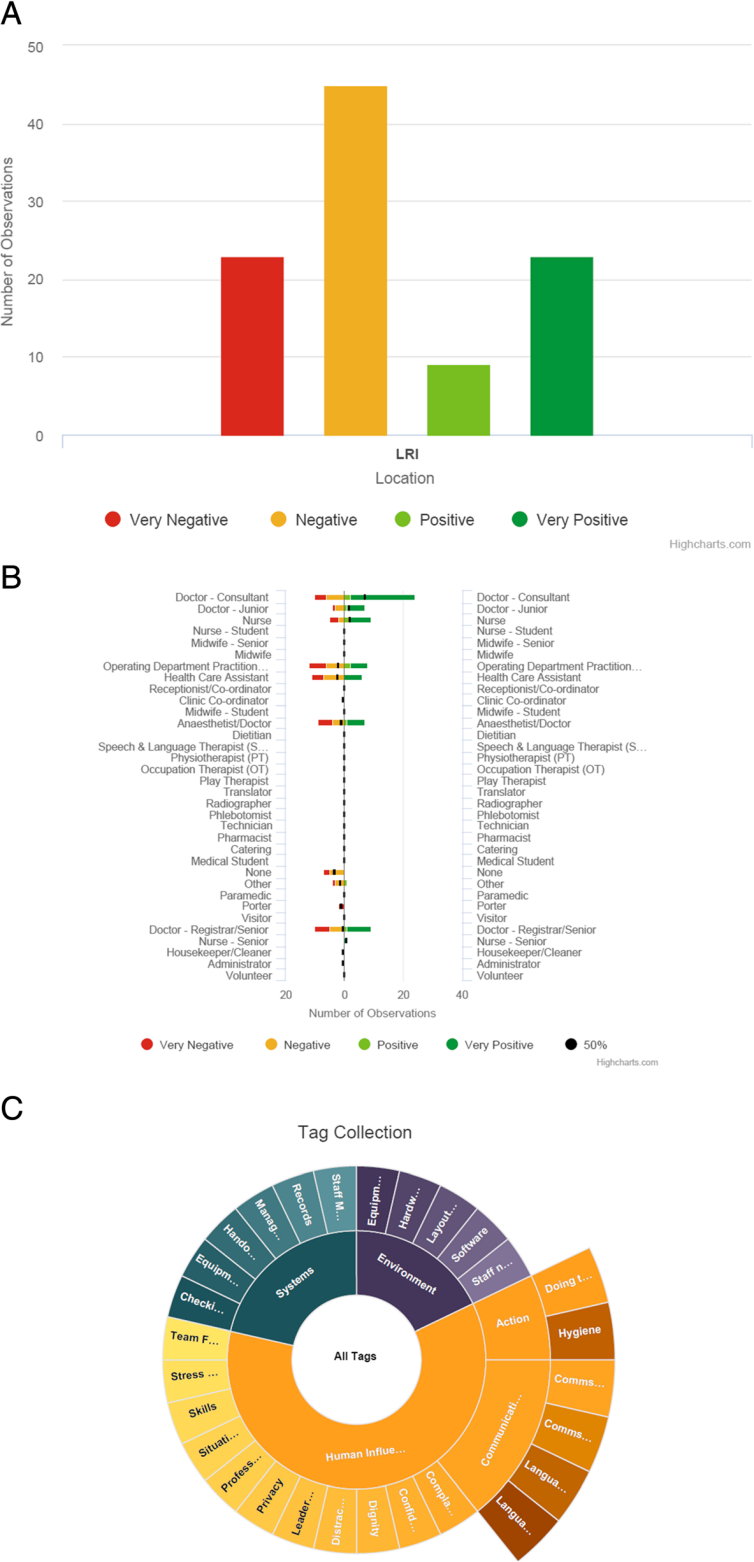
**A** Data from iPad Observations in 2019. **B** 2019 Observations — observed practitioner. **C** Chart showing how to also present the 2019 data using all tag headings

A total of six final-year medical students worked with the new app using iPads in a range of clinical areas (again theatres, clinics and wards) in the same hospital. Ahead of the final SSM, two students used and tested the app in December 2018 and working individually made 28 observations in 2 half-days. The remaining four final-year medical students were trained to use the new app in June 2019. They each spent 3 half-days and made a total of 72 recordings. Together, these findings totalled 100 observations of which 68 were negative and 32 were positive. The majority were again relating to human influences. The app shows these outcomes in a variety of different ways (Fig. 3A–C).

#### Evaluation of stakeholder perspectives

Stakeholder perceptions were gained from five final-year medical student ‘observers’ and 11 clinical staff members ‘observed’. These were doctors of various grades, scrub nurses, advanced nurse practitioners and nurse ward managers. The data are presented as themes and extracts (Table 7).

**Table 7:** Qualitative excerpts

Theme	Interview ixtracts
***The value of the observation method for learning***	**Clinical team learning (sub-theme)** • ‘...you can also then look at areas of good practice and areas of poor practice and see is it that the areas of poor practice can learn from areas of good practice, because these observers can pick up areas of good practice as well. So the good thing is that they would highlight both, you can then compare and say why is it that it works well in one setting and a similar setting in another specialty, why doesn’t it work so well?’, H005. • I think it would be a good thing. I think it’s one of the most important things that we can help with, patient safety, and I think it helps us to realise areas where there’s potential to improve things. I think a lot of us are aware of things that could be improved but I think a lot of us aren’t aware of other things that other people might pick up. H006 • ‘I think that’s what we need to sort of like focus on because for too many years, it’s always been very negative. And we need to highlight there’s a lot of people out there doing a fabulous job, and they aren’t highlighted enough, and that work isn’t highlighted enough. And that’s not me saying we don’t need to look at the things that need to be improved, because that’s essential, but we don’t praise where praise is due. H009 **Student learning (sub-theme)** ‘I think it makes you more vigilant in your own practice when you have to observe other people making those mistakes. So things like washing hands or putting patient detail away or something like that, like I feel like I’m more aware of myself in those settings because I’ve had to observe someone else.’ S004
***Acceptability and impact of the observation process***	**Acceptable to be observed: staff/practitioner** • ‘The consultants weren’t that bothered… they’d start to have comments like ‘oh, patient safety whatever’…it was more the nurses and the ODPs who were a bit more like ‘oh my God, we have to be careful’. S004 • ‘I wasn’t aware that I was watching different grades. I mean obviously I know I am but I wasn’t, I didn’t feel any different making observations from the consultants than from the HCA [Health Care Assistant] type thing’, S001. **Acceptability of being the observer: students** • ‘…it felt in some ways like we were almost marking them which it’s sort of the paradigm…that’s essentially what it’s done to us, if someone’s standing there with a checklist, they're marking you and you can pass or fail… so there was that feeling of trying to accommodate for that, being almost overfriendly.’ S002. • ‘…so I asked ‘are you happy for me to be around?’ and they said yes, but then I could clearly tell that they weren’t and kept making comments about the fact that I was there and I was watching– I mean obviously it was difficult for me being in that situation. I didn’t find it difficult for me but more for the other clinician she was working with because I think she was trying to sort of, she wanted someone to back her up and they were kind of ‘well no, it’s fine that she’s there’, so I could feel that there was a bit of tension there.’ S001. • ‘I felt very awkward because I could sense the anxiety and the discomfort surrounding my observations, especially when I used to put pen to paper, everyone used to kind of just like tense up and bit and then – then they wander around trying to see what I had written….they were quite confrontational, it was just like ‘what have you noticed?’ or ‘what are writing?’ So they said to me they didn’t feel comfortable with me writing things.’ S004. **Concerns for being observed: staff/practitioner** • ‘…with someone that observes, you always associate that with being critiqued…for someone else to watch you do it it’s more nerve racking because you’re thinking ‘oh my God’ you’re double guessing what you would normally do in practice… And I think sometimes you’re more likely to make errors because you’re nervous.’ H008, Nursing staff. • ‘Well personally I’m very used to it, having medical students in clinic sitting behind me, so that wasn’t an issue. But I think I was more aware of what I was doing. I think it formalised me a little bit.’ H001, Doctor. • ‘…but it is a little bit nerve racking at times, you do second guess yourself and double check. But to be fair they were unobtrusive and I never felt, whether I was observed, I never really knew whether I was observed or not.’ H007, Nursing staff. **Concerns for being the observers: students** • ‘I felt like I think it was the lack of prior notice that made them feel a bit more like they were being watched. Because obviously when you say patient safety I think what they assumed would be human factors down to them which made them more hostile. There’s no other word for it really, or nervous. And so when, because one of the categories was equipment failures or shortages or understaffing and when I focused more on those things they were a lot more open about the struggles they’re facing, because they felt like they were going to make a difference.’ S004
***The process of anonymity of observations***	**Staff views on anonymity** • ‘I presumed it was anonymous, however if they saw something that was particularly unsafe, you know, anywhere I would have hoped that they would have raised it and felt confident to raise it on the day rather than just to write it down that there was a problem.’ H002 • ‘I mean if nothing else, if it was all positive, it helps form part of your sort of professional development portfolio, it would help that, but actually if it raises some good points along the way and having it quite independent because a lot of people in their professional development portfolios, you know, it’s fairly biased, I would argue. And actually having an independent student who would look at things slightly differently is useful I think’. H004
***The mechanism for recording observation***	**Staff views** ‘At the time they had paper but they’re now trialling an electronic form. Which I think may have pros and cons. I think an iPad is quite acceptable. If they’re doing it on their phone I think people think that they’re texting and things like that. So phones don’t go down very well…An iPad I think is generally more accepted as a learning tool. All the students now have iPads and it’s more thought of if you’re on an iPad that you’re actually working’.H006 **Student views** ‘I think in some ways it [the iPad] is more discrete. As students we have our iPads with us on placement anyway so it’s not as obvious what we’re doing. I think the clipboard itself can be quite threatening and yeah, so I think the iPads are a good idea’. S003
***The use of the observation data post-collection***	**Staff views** • ‘I think if it raises some points, you know, generally about things that people are doing subconsciously – or not doing subconsciously – and actually I appreciate it’s anonymised but actually if someone’s able to turn round and say ‘do you realise you do this?’, or don’t do this, that’s actually quite helpful.’ H004 • ‘I would personally want it fed back to myself as a Ward Sister. Now I have responsibility for this ward so if the practice is not of an expected standard I would like to know about that personally so I could disseminate it back to my team’. H009 • ‘On the day, at the debrief, yeah, that would be great. I think that would be helpful in that situation rather than later on, when you’ve forgotten about it.’ H010 • ‘We have briefings at the beginning of every list and if anything changes, so if we have different staff in the afternoon to the morning we have another briefing before the beginning of the afternoon list…yeah, there’s plenty of time to feed it back. If it’s something that’s sort of happened during that procedure that they’ve noticed and they gave us feedback straight away, you can feed it straight back in the debrief. But if it was more that they wrote it down on their iPad and handed it in, then obviously by the time the next debrief it will be a completely different team. So more of like the trends of what they see over the two days of observing. I think we tend to have them once a month and also our consultants have a meeting as well. We also have like audit quality improvement [0:08:21] so that would be another place where students could actually come to that and present it themselves’. H006

*The value of the observation method for learning* was confirmed by both the students who were observing practice and the observed practitioners. *Front-line practitioners* perceived the value of the recordings to enhance individual and team learning in clinical practice. For the clinical teams, the work was perceived as a supplement to existing data, such as safer surgery theatre checklists and clinical audits, because it could record a wider range of habitual practices and take account of environmental factors. It was felt that the observation process captured both good and poor practice and that this was helpful for teams in implementing appropriate improvements. The observed practitioners referred to ‘a climate of negativity’ around patient safety and praised the data for allowing the recording of positive clinical practice to provide both balance and an accurate representation of everyday practice. This was something they felt was lost with other recording practices which focused solely on poor practice. Shared learning across clinical areas was discussed as advantageous, particularly the ability to learn from areas showing excellence (Table 7).

Senior medical students perceived this as a good method for *student learning* on patient safety as they were forced to now see the totality of practice. As observers making the recordings, they recognised that this process helped them to reflect on how to take on an active role within a clinical team. Several students who had qualified by the time they were interviewed described how their observations had fed into their plans for improving their practice.

*Acceptability and impact of the observation process* was discussed by front-line staff and students. The majority of *practitioners* were happy to be observed and confirmed that it was *acceptable to be observed*. *Students* felt equally comfortable observing all levels of staff grade. There were *some concerns* from mainly non-medical *practitioners*, who spoke about feeling additional pressure and being suspicious of being watched (Table 7). At the start of the observations practitioners being observed displayed a kind of Hawthorne effect, with the presence of observers affecting the practice of those observed. They seemed to follow protocols more closely and displayed exemplary behaviour, for example, taking more time than usual to introduce themselves and team members to patients. After a little while, all staff quickly returned to practising unaware of being observed because they were busy. The medical students sensed the tensions and described adopting a friendly persona to gain practitioners’ confidence and assert their position as helpful observers. Some students eased tensions by reminding observed clinical staff that they were ultimately looking at what they did as individuals but also within the systems and the environment where they were working.

Observed staff commented on the way the students introduced the work prior to commencing their observations. Some staff were not informed and unaware that observations were taking place as an exercise for patient safety and received limited information and, therefore, they felt threatened. In these situations, students had to re-explain the project’s purpose and the anonymity of the process to ease concerns. Some students defused tensions by offering informal feedback afterwards with everyone on the observed team. This, too, appeared to allay concerns. The acceptability of the observations greatly increased with clarity about the reasons behind the process.

*The anonymity of observations* was a strong theme*.* All staff were aware that observations were anonymised and valued this, but some were not convinced this was the way forward. In these instances, practitioners wanted the observer to draw attention to malpractice, either by overstepping the line of ‘observer’ by intervening in real-time in the situation, or by having permission to report the action(s) after the event. Such an approach would, of course, mean that observations had the potential to have negative consequences to individual staff members, rather than being used to identify higher-level trends across departments. It was also suggested that observations featuring good practice might support doctors training portfolios by providing specific objective examples of their work.

*The use of the observation data post-collection* revealed that staff wished to receive information from the observations as soon after data collection as possible due to shift patterns and the rotation of staff and for immediately learning. Those requesting personal feedback on their individual performance also asked for this immediately after the observation session. Daily briefings conducted by teams were signalled as a place for rapid feedback to be shared and in this way staff felt any required changes were more likely to be implemented. This was compared to the delayed trust information distribution in the form of emails and bulletins. Monthly team meetings were mentioned as a means of reinforcing information given during daily briefings as well as the appropriate environment for reflecting on data trends over time.

*The mechanism for recording observations* revealed a strong preference for the use of the electronic recording device. Students and staff who had experienced both paper and app recordings commented on their preferences. Visually, being seen with a clipboard was described as off-putting. In contrast, observers and observed staff overwhelmingly favoured the electronic device, as these were now familiar to patients and clinicians within clinical environments and, thus, both acceptable and inconspicuous.

## Discussion

Since 2002, expert observers on normal flights have collected data about flight crew behaviour, as threats or errors, through an approach known as line operations safety audit (LOSA). The collective findings are fed back into practice and continue to support improvements [35, 36]. Today, these audits are conducted within a strict no-jeopardy context: in other words, flight crews are not held accountable for their actions or errors that are observed [43]. The observers know the procedures and checks thoroughly. During flights, the observer records how flight crews manage these errors and specific human behaviours associated with accidents and incidents. LOSA can be used at any time and the deidentified findings result in learning and efforts to improve performance. In seeking to offer medical students deeper insights into safe practice, we set out to design observational learning, adapting the aviation LOSA methodology for students to experience the complexity of being a member of a healthcare team in an acute hospital.

Our students were initially tasked with looking for poor practice but told us they were also drawn to identify good practice. The final product, a recording app entitled Healthcare Team Observations for Patient Safety (HTOPS), has the potential to pick up ‘light noise’, what is actually happening in real time, using an anonymous feedback system to record poor and excellent practice. The app presents a novel, non-threatening mechanism to identify low-grade risks to patient safety, while providing active learning on patient safety for medical students. We have evidence that students left their special study module with richer and deeper appreciations of everyday human foibles and weaknesses and possibilities for excellence, as soon to be members of care teams.

Teaching tomorrow’s practitioners about safe practice, despite helpful directives [25, 26], remains daunting. There are many social and psychological theories on human behaviour [44–48], challenges for whole cohort engagement with Quality Improvement techniques [49] and huge amounts of time and resources can be spent on poorly constructed simulations [50]. Within the undergraduate curriculum, we are experimenting with how to distil complex concepts and help inexperienced undergraduates appreciate the expansive levels of knowledge on patient safety. Many have perceived that students and junior staff can become the eyes and ears of an organisation, but as yet we have not harnessed this or considered using this as a teaching method, which enables students to become partners in propelling and contributing to optimal practice.

Our first challenge was to apply aviation observation methods to student learning within the complex systems of healthcare delivery. We sought to avoid designing yet another self-reported measurement tool, as there is a strong acknowledgement that healthcare staff feel overburdened with form-filling tools and yet require a voice [51, 52]. Using participatory action research, supported by a steering group with wide representation, ensured that our cyclical data was debated and discussed so that we moved from aviation thinking to healthcare thinking iteratively. Student willingness to learn more about patient safety using the medical school special study route yielded active participants, while the local acute hospital was more than willing to engage as partners in the project. The direction of travel was aided by an IT technologist seeking a simple yet workable solution, which required several paper prototypes before being applied to an app. The final product was found to be quick and easy to use and students could quickly enter and code both positive and negative observed patient safety behaviours.

Acceptability within clinical areas for student observations, despite team consent to be part of the research, proved challenging. Some clinical practitioners welcomed being observed, while others were sceptical. Expecting qualifying students to explain their presence to unprepared seniors brought some concerns, although the students appeared to be confident to show and share the potential of the actions. Anonymity was paramount to acceptance because of avoiding a blaming of others culture, though some interview participants noted that this could limit the usefulness of feedback in making changes. Overcoming cultural challenges in healthcare and moving from the status quo remains a concern for patient safety leaders and will apply here, as ‘trust’ with this process remains paramount. There was a desire for instant feedback. This was because several leading clinical nurses and doctors could perceive this as a vehicle to help them advance good practice, not only because it picked up ‘light noise’ but also because it could highlight positive actions.

The final thematic analysis resonates with others who have tried to map the breadth and depth of contributory factors for patient safety [4]. At this stage, we do not claim this is complete and the iterative nature of this development allows for on-going development and refinement. An essential responsibility for the usefulness of the intervention, however, lies with the observer as they can write and clarify what they observe, offering not just factors but real-time stories revealing more about the context. The next steps require further studies to (i) confirm the app is complete and workable, (ii) develop a training and learning event for all observers, (iii) explore the potential integration into everyday practice in clinical areas, and (iv) affirm whether this changes practice and whether areas where possible poor practice is repeatedly identified do reflect and take this feedback on board. Finally, (v) we need to confirm whether practitioners trust and are willing to invest in this method. It is possible to expand the observer role to include qualifying nurses and allied health practitioners and to qualified staff. The advantage here is that becoming an observer as a student appears to heighten the desire to be a good practitioner. Being a qualified observer, therefore, could offer a chance for more senior practitioners to learn about and deepen their understanding of the theory of human behaviour in groups and human fallibilities. The concern is that deploying qualified staff as observers may evoke more suspicion amongst those being observed and that the methods would lose some of the naivety because of normalisation. On the other hand, this might be better for speciality-specific safe practice.

This early pilot study has *limitations.* The small set of students were a self-selecting group of final-year students who were keen to know more about patient safety. It is difficult to distinguish between their desire to increase their knowledge and actual commitment to observe seniors in real-time clinical situations. Despite this, three different groups of students all engaged with the project and produced comparable results. Much depended upon the thematic analysis of what was observed as students went onto the wards, and the final app will require further work and further testing to reach a more complete set of possible activities to be scored. Interviews with students and front-line staff were often compromised through participant availability within busy student and clinical working schedules.

## Conclusion

Patient safety remains a crucial challenge for modern healthcare delivery. Retrospective analysis of what goes wrong is now being aligned with real-time considerations for adaptive practice that ensures forward thinking [16]. HTOPS offers a testable approach for learning. The process requires further study but this pilot data offers possible solutions not just for highlighting light noise but for deeper learning about safety because the process has the potential to link theory to practice.

## Data Availability

Availability of data and materials are in the main shown in this document as they are contained within the tables. The tables summarise the paper reports of the medical students which are stored at the University of Leicester in a locked cupboard and are available for reference on request. The final set of data is available on the app on request for which we share a summary in the paper.
